# Cerebro-Spinal Fever

**Published:** 1905-09-02

**Authors:** 


### Cerebro-Spinal Fever.

In our issue of April 15 we ventured to express the opinion that the outbreaks of epidemic cerebro-spinal meningitis reported from America and from Central Europe had certain dangerous possibilities for Great Britain, and we then urged that the sanitary authorities should keep a specially keen outlook for the appearance of cases of the disease in our midst. It is, therefore, with considerable satisfaction that we take note of the circular just issued by the Local Government Board to borough councils and other local health authorities. The facts, so far, are entirely reassuring, as there is no reason whatever to believe that at the present time the disease is more prevalent in this country than it has been from time to time during the last quarter of a century. Indeed, it is one of the events to be chronicled and studied in the natural history of cerebro-spinal fever that, though occasional sporadic cases have been noted in the United Kingdom, the disease here has never attained the epidemic prevalence which has frequently marked its career in certain other countries. Upon what influence or factor this relative immunity depends is quite uncertain, and hence we have no guarantee of its endurance. Thus it is all to the good that the chief authorities should officially warn those who are immediately in charge of the public health of possible dangers ahead, and should indicate how these dangers may best be avoided.

With the circular of the Board is included a memorandum prepared by their medical officer, Dr. W. H. Power, and in this the various aspects of the disease are set forth in a very interesting and practical fashion. Particular stress is laid on the milder and anomalous forms in which cerebro-spinal fever not infrequently appears, and which are not only apt to be overlooked but may readily be mistaken for other maladies, such, for example, as enteric fever, influenza, and pneumonia. As in other diseases of epidemic character it is these milder cases which carry the maximum of risk to the community, and it is proper, therefore, that emphasis should be laid on their occurrence. There is, indeed, no room for doubt that not only single cases, but even groups of cases, of cerebro-spinal fever have been, so far as their true nature is concerned, entirely overlooked, and the outbreak has been inadvertently concealed under one or other of the diagnostic terms to which allusion has just been made. The expectation of a more satisfactory result at the present date, should similar circumstances recur, rests upon two grounds. First, there is an increased recognition of the possibility of these atypical cases of the disease and of the ease with which the true diagnosis may be missed; towards this recognition a distribution of Dr. Power's memorandum will be a further help. The second aid in the accurate diagnosis of cerebro-spinal fever is provided by the progress of bacteriology. Symptoms, as indeed the medical profession knows only too well, may be anomalous or even misleading, being, as they often are, coloured and refracted by the personal idiosyncrasies of the patient. But when the diagnostic inquiry can push its way into the objective evidence, and can search for a precise and specific cause, the hope of a certain, definite, and confident result is enormously increased. Hence it is satisfactory to note that lumbar puncture and a search for the diplococcus meningitidis intracellularis in the cerebro-spinal fluid are insisted on in the official memorandum as forming an important aid to diagnosis. Not less welcome is the intimation that the Local Government Board will be prepared on the reception of a report from the local medical officer through the sanitary authority for the district to afford such advice or assistance as, in the circumstances, may appear to be necessary. The Board, also, will be prepared to consider applications for the extension of the provisions of the Infectious Diseases (Notification) Act to the case of cerebro-spinal fever. On this point we should be disposed to suggest that such applications should not be delayed until what the circular calls "special circumstances" arise. That occasional cases of the disease occur is certain. In this country, fortunately, they seldom or never, for some reason or other, form the starting-points for an epidemic outbreak. But it is in the public interest that they should be recognised and recorded, and the official machinery necessary to secure this should be put into operation without delay. As the Local Government Board's circular and the memorandum of the official medical officer will help to secure these ends they are to be heartily welcomed, but we think a policy of compulsory notification ought at once to be inaugurated.

